# Upregulation of Insulin and Ecdysone Signaling in Relation to Diapause Termination in *Bombyx mori* Eggs Exposed to 5 °C

**DOI:** 10.3390/insects15120989

**Published:** 2024-12-12

**Authors:** Shi-Hong Gu, Pei-Ling Lin

**Affiliations:** Department of Biology, National Museum of Natural Science, 1 Kuan-Chien Road, Taichung 404, Taiwan

**Keywords:** insulin, EPPase, ecdysteroid, embryonic diapause, diapause termination, bombyxin, chilling

## Abstract

In the present study, we investigated the possible correlation between insulin/ecdysone signaling and chilling-induced egg diapause termination in *Bombyx mori*. Changes in *insulin* (*bombyxin-Z1*) and *ecdysteroid-phosphate phosphatase* (*EPPase*) gene expression levels in chilled eggs (whose diapause had been terminated by chilling to 5 °C for 90 days) exhibited no significant increase after being transferred to 25 °C, which differed from both non-diapause eggs and HCl-treated eggs. We further compared the differential temporal expressions of *insulin* (*bombyxin-A6*, -*Y1*, and *-Z1*), *ecdysone signaling* (*EPPase* and *E75A*), and *metabolic-related* (*trehalose transporter 1* (*Tret1*) and *trehalase 1* (*Treh1*)) as well as *sorbitol dehydrogenase 2* (*SDH2*) genes between chilled eggs and eggs kept at 25 °C. Our results showed that all gene expressions remained at very low levels in eggs kept at 25 °C. However, in chilled eggs, differential temporal changes were detected according to different genes, with *bombyxin-A6* and *EPPase* gene expression levels being maintained at relatively constant, high levels. Higher expression levels of the *E75A*, *Tret1*, and *Treh1* genes were also detected in chilled eggs. Expressions of the *SDH2* and *bombyxin-Z1* genes decreased during the first 15 days and then increased between days 30 and 90 of chilling. Ecdysteroid levels and phosphorylation of glycogen synthase kinase (GSK)-3β, a downstream target of insulin signaling, were maintained at relatively higher levels in chilled eggs. These results suggested that due to relatively higher insulin and ecdysone signaling levels in chilled eggs, relatively higher glucose metabolism was sustained, leading to the continued depletion of metabolic reserves. On day 30 of chilling, as a means to adjust nutrient requirements and maintain embryonic nutrient homeostasis, *SDH2* gene expression began to increase, followed by increased expression of the *bombyxin-Z1* gene. Along with high expressions of the *bombyxin-Z1* and *SDH2* genes, a decreased sorbitol level was suggested to eventually terminate diapause in *B. mori* eggs. To our knowledge, this is the first study to demonstrate the correlation between insulin/ecdysone signaling and chilling-induced embryonic diapause termination.

## Simple Summary

Few studies have been conducted on the signaling mechanism underlying chilling-induced embryonic diapause termination in insects. In the current study, we compared temporal changes in expression levels of *insulin* (*bombyxin-A6*, *-Y1*, and *-Z1*), *ecdysone signaling* (*ecdysteroid-phosphate phosphatase* (*EPPase*) and *E75A*), *sorbitol dehydrogenase 2* (*SDH2*), and *metabolic-related* (*trehalose transporter 1* (*Tret1*) and *trehalase 1* (*Treh1*)) genes between chilled *Bombyx mori* eggs and eggs kept at 25 °C. Our results showed that expressions of all the above-mentioned genes remained at very low levels in eggs kept at 25 °C. However, in chilled eggs, differential temporal changes were detected according to different genes, with *bombyxin-A6* and *EPPase* gene expressions being maintained at relatively constant, high levels. Expressions of the *SDH2* and *bombyxin-Z1* genes decreased during the first 15 days, and then *SDH2* gene expression began to increase on day 30 after chilling, followed by increased expression of the *bombyxin-Z1* gene. Relatively higher GSK-3β phosphorylation and ecdysteroid levels were also found in chilled eggs. From these results, we suggest that the upregulation of insulin/ecdysone signaling in chilled eggs is related to chilling-induced egg diapause termination in *B. mori*.

## 1. Introduction

To survive winter periods, insects living in temperate zones undergo diapause, a state of developmental arrest [1,2,3]. The silkworm *Bombyx mori* is a typical insect species that undergoes embryonic diapause [4,5,6,7,8]. A diapause hormone, which is secreted from suboesophageal ganglia during the pupal period of the mother moth, appears to act on her developing ovaries [4], resulting in embryonic diapause in the next generation. For diapause-destined embryos, cell division is arrested, and embryogenesis ceases during the G2 cell cycle stage immediately after the formation of the cephalic lobe and telson and sequential segmentation of the mesoderm [9]. As long as eggs are incubated at 25 °C, a diapause state is maintained. In contrast, if diapause-destined eggs (approximately 20-h-old eggs) are treated with an HCl solution (specific gravity 1.075 at 15 °C) for 5 min at 46 °C, the entrance of diapause is prevented, and eggs continue to undergo embryonic development until larval hatching. Chilling is generally effective in terminating embryonic diapause. Exposure of diapausing eggs to 5 °C for 2~3 months completely terminated diapause, and the eggs resumed embryogenesis after being transferred to 25 °C [5]. Non-diapause eggs from the polyvoltine strain continued embryonic development at 25 °C. When developing eggs (non-diapause eggs, HCl-treated eggs, and chilled eggs) were incubated at 25 °C, eggs hatched on about days 9 or 10.

Several studies were conducted on the mechanism underpinning diapause termination. Sorbitol and ecdysteroid metabolism appear to be involved in regulating diapause termination [5,10]. Diapausing eggs contain substantial amounts of sorbitol and glycerol, which are converted from glycogen [11]. It was further shown that sorbitol is a strong factor in arresting embryonic development, and once sorbitol surrounding the embryo is removed, development can proceed [12]. During the diapause termination process in chilled eggs, sorbitol is reconverted to glycogen, and this reaction is regulated by NAD-sorbitol dehydrogenase (SDH) [13,14,15]. This enzyme activity parallels those for amounts of protein and mRNA, indicating that the *SDH* transcription level plays a role in regulating enzyme activity [16,17]. A detailed investigation showed that *SDH* gene expression increased after 40–50 days in chilled eggs, indicating a correlation between expression of this gene and initiation of diapause termination [16,17]. Later studies demonstrated a correlation between mitogen-activated protein kinase (MAPK)/extracellular signal-regulated kinase (ERK) signaling and the diapause termination process of *B. mori* eggs [18,19,20,21]. It was found that in diapause eggs incubated at 25 °C, ERK phosphorylation was maintained at a low level. However, in chilled eggs, a high ERK phosphorylation level was detected in yolk cells after 45 days of incubation at 5 °C. It was further shown that activation of ERK phosphorylation appeared to regulate yolk cell granulation and dissociation, which began at the same stage, indicating that ERK signaling plays a role in regulating diapause termination of *B. mori* eggs [18,21]. ERK signaling was also found to be related to expressions of *SDH2* and *ecdysteroid-phosphate phosphatase* (*EPPase*) genes [21]. However, no information has focused on detailed temporal changes in *EPPase* gene expression in chilled eggs, and few studies have been conducted on the signaling mechanism underlying chilling-induced diapause termination.

In addition, numerous studies pointed to the insulin signaling pathway as a possible mediator of nutrient sensing and uptake [3]. Insulin/insulin growth factor (IGF) signaling appears to regulate metabolism, growth, development, and aging [3,22,23,24,25,26]. Our previous study of *B. mori* showed that different expression levels of glycogen synthase kinase (GSK)-3β phosphorylation (a downstream signaling target of insulin) exist between diapause and developing eggs [27]. We found that with the onset of diapause, an abrupt decrease in GSK-3β phosphorylation levels occurs. However, in developing eggs (both non-diapause eggs and eggs whose diapause initiation was prevented by HCl), levels of GSK-3β phosphorylation appeared to remain relatively high for several days and then greatly decreased 2 or 3 days before hatching, clearly indicating that insulin signaling is related to the diapause process in *B. mori* eggs [27]. Moreover, we found significantly different temporal changes in GSK-3β phosphorylation levels between chilled eggs and eggs kept at 25 °C. In eggs exposed to 5 °C, relatively high levels of GSK-3β phosphorylation were detected. In contrast, in eggs incubated at 25 °C, GSK-3β phosphorylation levels were very low [27]. However, the biological functions of these temporal changes are not clear. Considering that GSK-3β phosphorylation plays an important role in insulin signaling [28], we hypothesized that differences in insulin signaling between chilled eggs and eggs kept at 25 °C may exist.

Considering that the detailed mechanism of how chilling treatment controls gene expressions remains to be resolved, we examined expression levels of several genes with different functions during the chilling period and compared them to those that were kept at 25 °C. We selected *bombyxin-A6*, *-Y1*, and *-Z1* as insulin signaling genes. *Bombyxin-A6* encodes bombyxin-II. The role of bombyxin-II, which is mainly produced by brain neurosecretory cells in *B*. *mori*, has been well documented [26]. We also recently reported that bombyxin-II stimulated expressions of the *sugar transporter* (*St*) and *trehalase 1* (*Treh1*) genes to enhance ecdysteroidogenesis in prothoracic glands [29]. *Bombyxin-Y1* is in a new class of insulin family peptides called the IGF-like peptides. It was previously demonstrated that *Bombyxin-Y1* is produced by fat bodies, the brain, and gonads, and its expression in fat bodies is stimulated by 20-hydroxyecdysone [30,31]. *Bombyxin-Z1* was previously reported to be related to embryonic development [32]. Considering that EPPase is an important enzyme that specifically catalyzes the conversion of ecdysteroid-phosphates to free ecdysteroids during embryonic development [33,34] and that E75A is an ecdysone signaling gene that appears to be related to silkworm embryonic development [35], we also examined expressions of these genes. SDH was reported to be related to the diapause termination process in chilled eggs [13,14,15]. It was shown that SDH2 is an SDH isozyme and that upregulation of both *SDH* and *SDH2* gene expressions and enzymatic activities in diapause eggs exposed to 5 °C were related to diapause termination [17,36]. We previously demonstrated that differential expressions of the *trehalose transporter 1* (*Tret1*) and *Treh1* genes exist between diapause and developing eggs [37]. Thus, we examined expressions of the above-described genes. Results of detailed temporal analysis of the above-described key gene expressions suggested that insulin/ecdysone signaling is involved in diapause termination of chilled eggs. 

## 2. Materials and Methods

### 2.1. Insects

Eggs of the silkworm *B. mori* (*Guofu* × *Nongfong*, univoltine strain) were used as diapause-egg producers. Larvae were reared on fresh mulberry leaves at 25 °C under a 12 h light (L): 12 h dark (D) photoperiod. Eggs laid during the first 3 h were pooled and then divided into two groups for the experiments. The first group of eggs was incubated at 25 °C under a 12 -L: 12 D photoperiod to maintain a diapause state. The second group was incubated at 5 °C in the dark after 2 days post-oviposition to terminate diapause [32,38]. When chilled eggs were transferred to 25 °C after a chilling period of 90 days, these eggs hatched on about day 9 or 10 after transfer. Diapause termination was confirmed by more than 95% hatchability. Detailed treatment schedules are described in the respective figure legends. Sampling was typically carried out by rapidly freezing experimental materials at −80 °C, and all samples were stored at −80 °C until use.

### 2.2. Reagents, Antibodies, and Enzyme Immunoassay (EIA) for Ecdysteroid Measurements

Anti-phospho-GSK-3β (Ser9) and anti-α-tubulin (α-tubulin) antibodies were purchased from Cell Signaling Technology (Beverly, MA, USA). A horseradish peroxidase (HRP)-linked goat anti-rabbit secondary antibody was purchased from PerkinElmer Life Sciences (Boston, MA, USA).

Ecdysteroids in chilled eggs (exposed to 5 °C for 90 days beginning at 2 days post-oviposition) and eggs incubated at 25 °C for 92 days post-oviposition were extracted with methanol and measured using a 20-hydroxyecdysone EIA kit (Cayman Chemical/Sanbio, Uden, The Netherlands) as previously described [29,39].

### 2.3. RNA Extraction and Quantitative Real-Time Polymerase Chain Reaction (qRT-PCR)

Total RNA was prepared from eggs (29 mg, about 50 eggs) using 600 μL of the TRI Reagent (Molecular Research Center, Cincinnati, OH, USA) according to the recommended protocol. The concentration was measured with a NanoPhotometer Pearl (Implen, Munich, Germany). RNA samples (1 µg) and a PrimeScript First-Strand cDNA Synthesis Kit (Takara Bio, Beijing, China) were used to synthesize the first-strand cDNA. 

Amounts of target gene mRNAs were quantified by quantitative real-time PCR (qRT-PCR), as described in previous studies [32,35]. The amount of *B. mori ribosomal protein 49* (*rp49*) was measured as an internal standard. The iQ5 Real-Time PCR Detection System (Bio-Rad, Hercules, CA, USA) was used according to the manufacturer’s instructions, and a melting curve analysis was applied to all PCR to ensure homogeneity of the amplified product. RT-PCR primers were designed according to parameters (no primer dimers and a product length of no more than 200 bp) outlined in the manual of the SYBR1 Green Real-time PCR Master Mix. The annealing temperature for all reactions was 59.5 °C. *C_T_* values were set against a calibration curve. The *ΔΔC_T_* method was used to calculate relative abundances.

It was reported that the *B. mori* genome contains 38 *bombyxin* genes, which are classified into 12 families of A to G and V to Z, and family A consists of 10 genes [26,40]. Considering that the roles of bombyxin-II in regulating silkworm growth and metabolism have been well documented and that *bombyxin-A6* and/or -*A7* encode bombyxin-II [26,41], we selected the *bombyxin-A6* gene to investigate its transcriptional regulation. However, due to the very high similarity in *bombyxin* A family members, primers for *bombyxin-A6* also recognize other *bombyxins* in this family including *bombyxin-A7* and -*A10.* The qRT-PCR was performed using primers listed in Table S1.

### 2.4. Western Blot Analysis

Sodium dodecyl sulfate (SDS)-polyacrylamide gel electrophoresis (PAGE) and Western blotting were described previously [27,38]. Eggs from the indicated days were homogenized in 10 µL of lysis buffer (10 mM Tris and 0.1% Triton X-100) at 4 °C [38], boiled in an equal volume of SDS sample buffer for 4 min, and then centrifuged for 3 min at 15,800× *g* to remove any particulate matter. Each 5 µL of supernatant (approximately 12 µg of total protein) was loaded onto 12% SDS gels. Gels were then transferred to polyvinylidene difluoride (PVDF) membranes using an Owl (Portsmouth, NH, USA) Bandit™ Tank Electroblotting System. Membranes were incubated overnight at 4 °C with primary antibodies and further incubated with an HRP-linked secondary antibody. The immunoreactivity was visualized by chemiluminescence using Western Lightning Chemiluminescence Reagent *Plus* from PerkinElmer Life Sciences. Films exposed to the chemiluminescent reaction were scanned and quantified using an AlphaImager Imaging System and AlphaEaseFC software, version 4.0.1 (Alpha Innotech, San Leandro, CA, USA).

### 2.5. Statistical Analysis

All data are presented as the mean ± standard error of the mean (SEM). Differences between groups were determined using Student’s *t*-test (for comparisons between two mean values) or by a one-way analysis of variance (ANOVA) followed by Tukey’s test (for comparisons between multiple mean values). Differences at *p* < 0.05 were considered significant.

## 3. Results

### 3.1. Temporal Changes in Gene Expression Levels of Bombyxin-Z1, EPPase, and Halloween (Spook (Spo)) After Chilled Eggs Were Transferred to 25 °C

**Figure 1:**
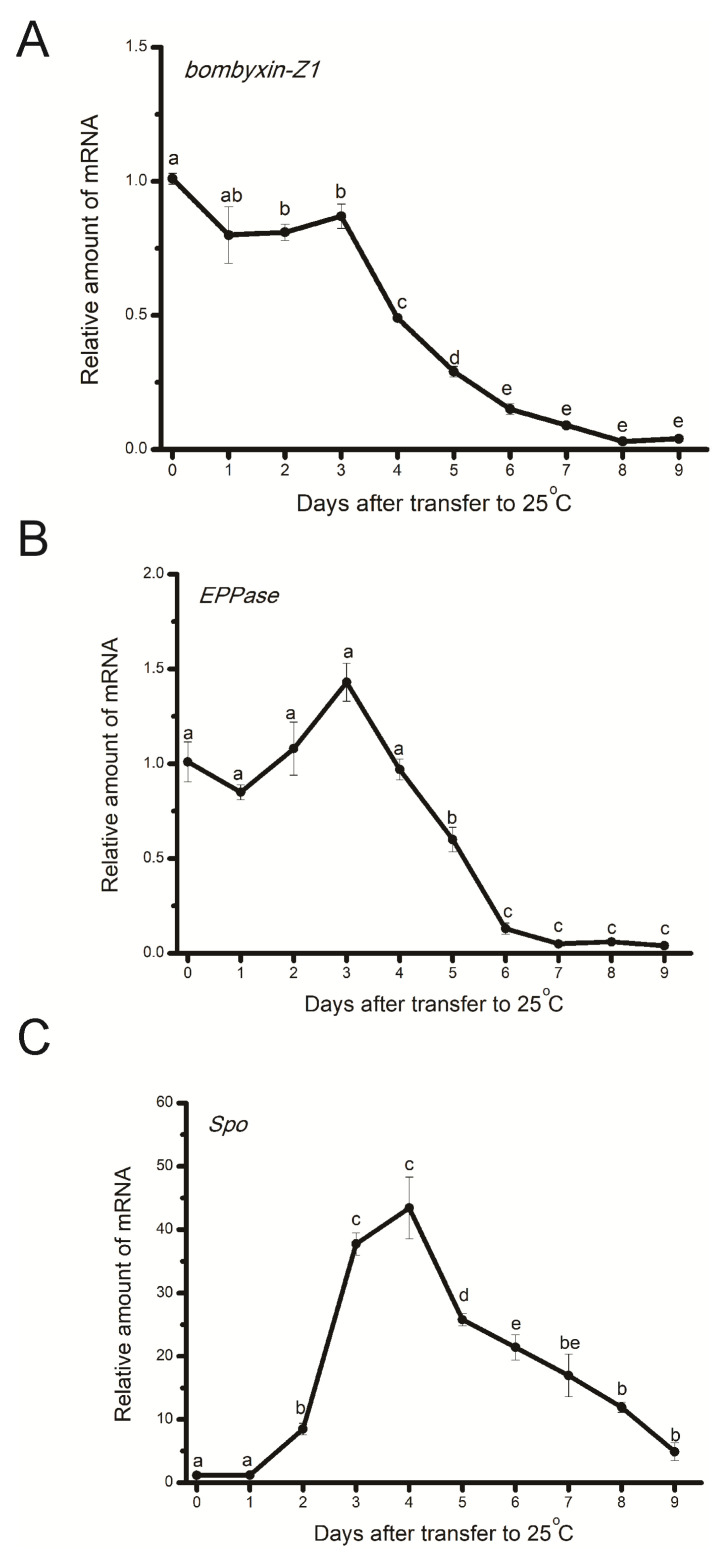
Changes in gene expression levels of *bombyxin-Z1* (**A**), *EPPase* (**B**), and *Spo* (**C**) in eggs in which diapause had been terminated by chilling at 5 °C for 90 days (chilled eggs). Egg extracts from each stage after being transferred to 25 °C were prepared, and gene expression levels were determined by a qRT-PCR. Gene expression levels of *bombyxin-Z1*, *EPPase*, and *Spo* relative to *rp49* were standardized to means of the first day after transfer to 25 °C. Each data point is the mean ± SEM (*n* = 4). Different letters above the bars indicate significant differences.

Our previous study showed that expression levels of the *bombyxin-Z1* gene in developing eggs (either HCl-treated or non-diapause eggs) exhibited later and broader peaks compared to those in diapause eggs during the early embryonic stage [32]. However, the changes in chilled eggs were not very clear. In the first experiment, we investigated changes in expression levels of the *bombyxin-Z1* gene in eggs in which diapause had been terminated by chilling at 5 °C for 90 days (chilled eggs) during embryonic development. As shown in Figure 1A, expression levels of the *bombyxin-Z1* gene were maintained at relatively higher levels on the first 3 days after being transferred to 25 °C. On day 4 and thereafter, levels decreased and reached a low level by day 9. This changing pattern greatly differed from both HCl-treated eggs and non-diapause eggs. We also examined changes in expression levels of the *EPPase* gene. As shown in Figure 1B, expression levels of the *EPPase* gene fluctuated during the first 4 days after transfer to 25 °C; levels had slightly increased by day 3 after transfer, and no further significant increase was detected. On day 5, a sharp decrease was detected, and very low levels were maintained between days 6 and 9. Expression levels of the *Spo* gene, a key ecdysone biosynthetic gene [42], showed different changing patterns, with a large peak being detected during the middle stage of embryonic stages of chilled eggs (Figure 1C). These temporal changes in *Spo* gene expression levels in chilled eggs were similar to those of both HCl-treated eggs and non-diapause eggs [35]. This result is consistent with a previous study showing that dephosphorylation of ecdysteroid is activated first, followed by the de novo ecdysteroid synthesis from cholesterol during the middle stage of embryonic development [34].

### 3.2. Differential Temporal Changes in Gene Expression Levels Between Chilled Eggs and Eggs Kept at 25 °C

The above results clearly indicated that expressions of the *bombyxin-Z1* and *EPPase* genes, but the not *Spo* gene, in chilled eggs showed no significant increase during the early embryonic developmental stages after being transferred to 25 °C, which greatly differed from those of both HCl-treated eggs and non-diapause eggs reported in previous studies [10,32,35]. This result indicated that before transfer to 25 °C, *bombyxin-Z1* and *EPPase* gene expressions, but not that of the *Spo* gene, might have undergone specific changes during the long chilling period, which resulted in differences in their expression patterns after being transferred to 25 °C compared to either HCl-treated or non-diapause eggs. This idea prompted us to further investigate whether differences in temporal changes in expressions of genes exist between eggs exposed to 5 °C and those incubated at 25 °C during the 90-day incubation period. Thus, we further examined changes in expression levels of several genes with different functions during the chilling period and compared them to those of the eggs, which were kept at 25 °C. 

**Figure 2:**
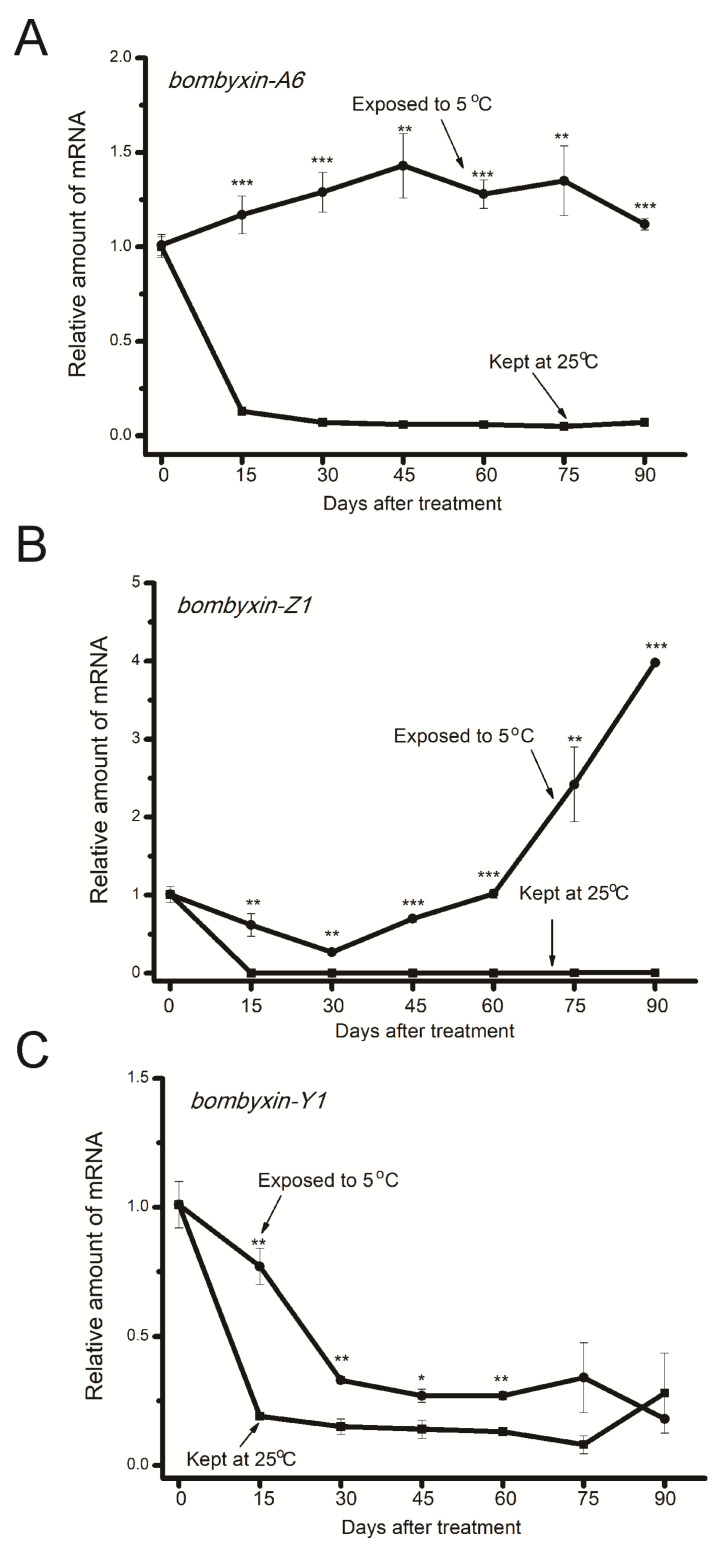
Temporal changes in gene expression levels of *bombyxin-A6* (**A**), *bombyxin-Z1* (**B**), and *bombyxin-Y1* (**C**) in diapause eggs exposed to 5 °C (circles) and those incubated at 25 °C (squares). After incubation at 25 °C for 2 days post-oviposition, diapause eggs were divided into two groups: one group was continually incubated to maintain diapause (squares), while the other group was exposed to 5 °C to break diapause (circles). Gene expression levels of *bombyxin-A6*, *bombyxin-Z1*, and *bombyxin-Y1* relative to *rp49* were standardized to the means of the day before transfer to either 25 or 5 °C. Each data point is the mean ± SEM (*n* = 4). Asterisks indicate a significant difference between eggs exposed to 5 °C (circles) and those incubated at 25 °C (squares) from the same day (by Student’s *t*-test, * *p* < 0.05, ** *p* < 0.01, *** *p* < 0.001).

As shown in Figure 2, upon exposure to 5 °C, *bombyxin-A6* gene expression was consistently maintained at relatively higher levels during the long chilling period compared to those at 25 °C. Temporal changes in gene expression levels of *bombyxin-Z1* during the first 30 days decreased in chilled eggs; on day 45 and thereafter, it increased and reached a high level by day 90. For *bombyxin-Y1*, similar changing patterns were detected in both chilled eggs and eggs incubated at 25 °C, although relatively higher levels were detected in chilled eggs on days 15 to 60.

**Figure 3:**
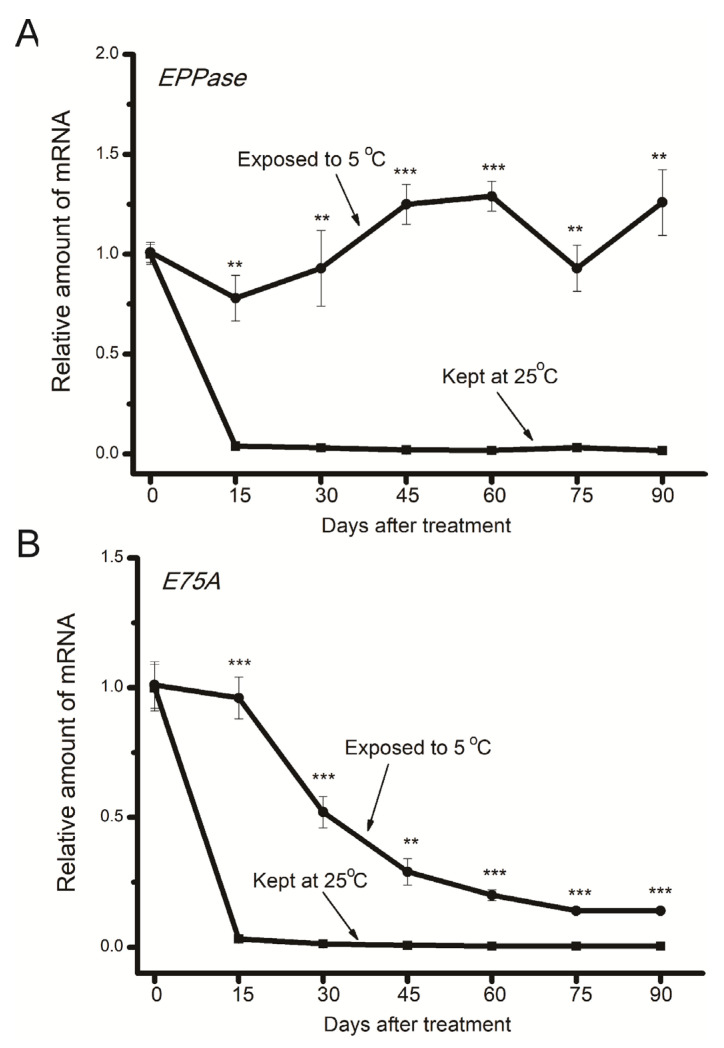
Temporal changes in gene expression levels of *EPPase* (**A**) and *E75A* (**B**) in diapause eggs exposed to 5 °C (circles) and those incubated at 25 °C (squares). Gene expression levels of *EPPase* and *E75A* relative to *rp49* were standardized to the means of the day before transfer to either 25 or 5 °C. Each data point is the mean ± SEM (*n* = 4). Asterisks indicate a significant difference between eggs exposed to 5 °C (circles) and those incubated at 25 °C (squares) from the same day (by Student’s *t*-test, ** *p* < 0.01, *** *p* < 0.001).

Figure 3 shows temporal changes in expression levels of the *EPPase* and *E75A* genes. Similar to *bombyxin-A6*, *EPPase* gene expression in chilled eggs was consistently maintained at relatively higher levels compared to those at 25 °C. Expression levels of the *E75A* gene were higher in chilled eggs, although gradually decreasing levels were detected in chilled eggs on days 15 to 45.

**Figure 4:**
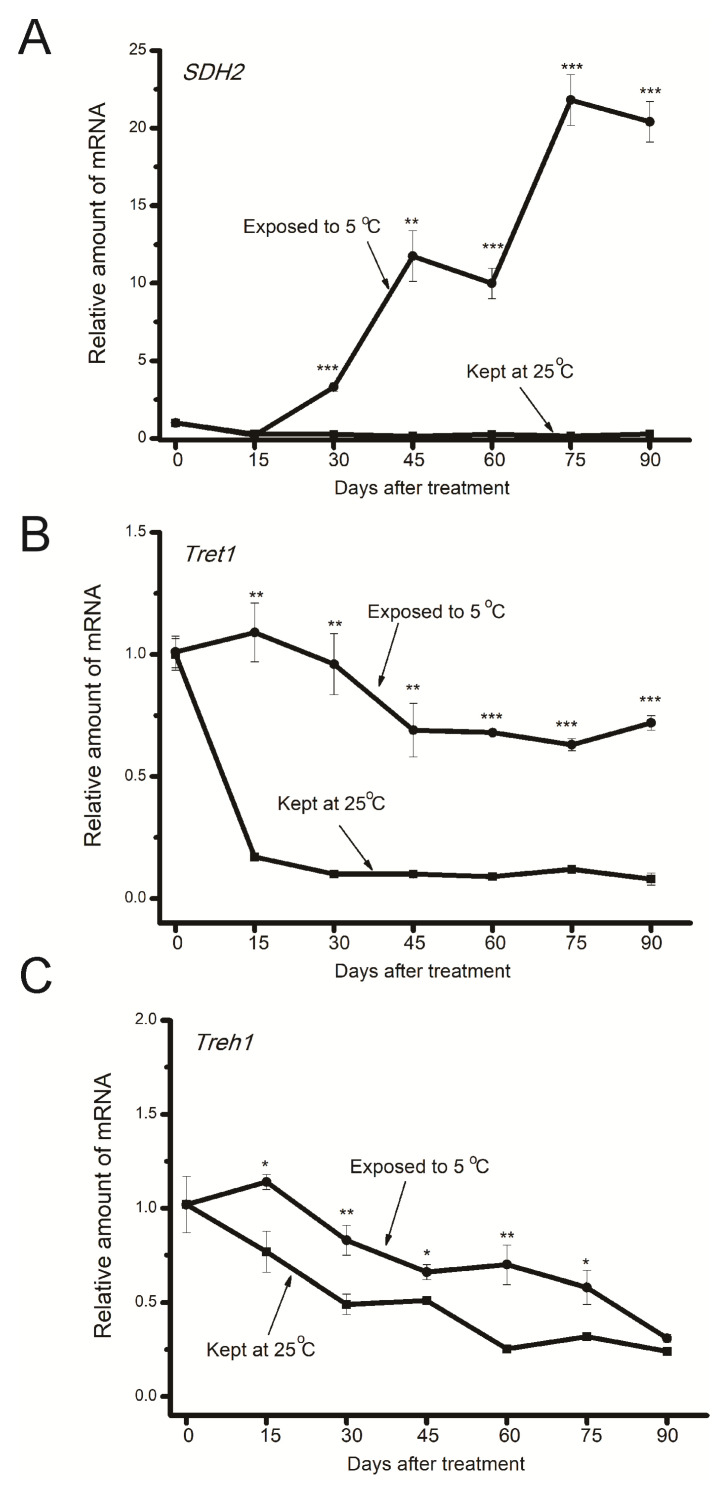
Temporal changes in gene expression levels of *SDH2* (**A**), *Tret1* (**B**), and *Treh1* (**C**) in diapause eggs exposed to 5 °C (circles) and those incubated at 25 °C (squares). Gene expression levels of *SDH2*, *Tret1*, and *Treh1* relative to *rp49* were standardized to the means of the day before transfer to either 25 or 5 °C. Each data point is the mean ± SEM (*n* = 4). Asterisks indicate a significant difference between eggs exposed to 5 °C (circles) and those incubated at 25 °C (squares) from the same day (by Student’s *t*-test, * *p* < 0.05, ** *p* < 0.01, *** *p* < 0.001).

Figure 4 shows temporal changes in expression levels of the *SDH2*, *Tret1*, and *Treh1* genes. *SDH2* gene expression level in chilled eggs during the first 15 days of the chilling period remained at relatively low levels; on day 30, the level began to increase and remained at high levels on days 45 to 90. The *Tret1* gene expression level was higher in chilled eggs compared to eggs incubated at 25 °C. Relatively higher *Treh1* expression levels were also detected in chilled eggs compared to eggs incubated at 25 °C except on day 90.

### 3.3. Effects of Chilling on Phosphorylation of GSK-3β

**Figure 5:**
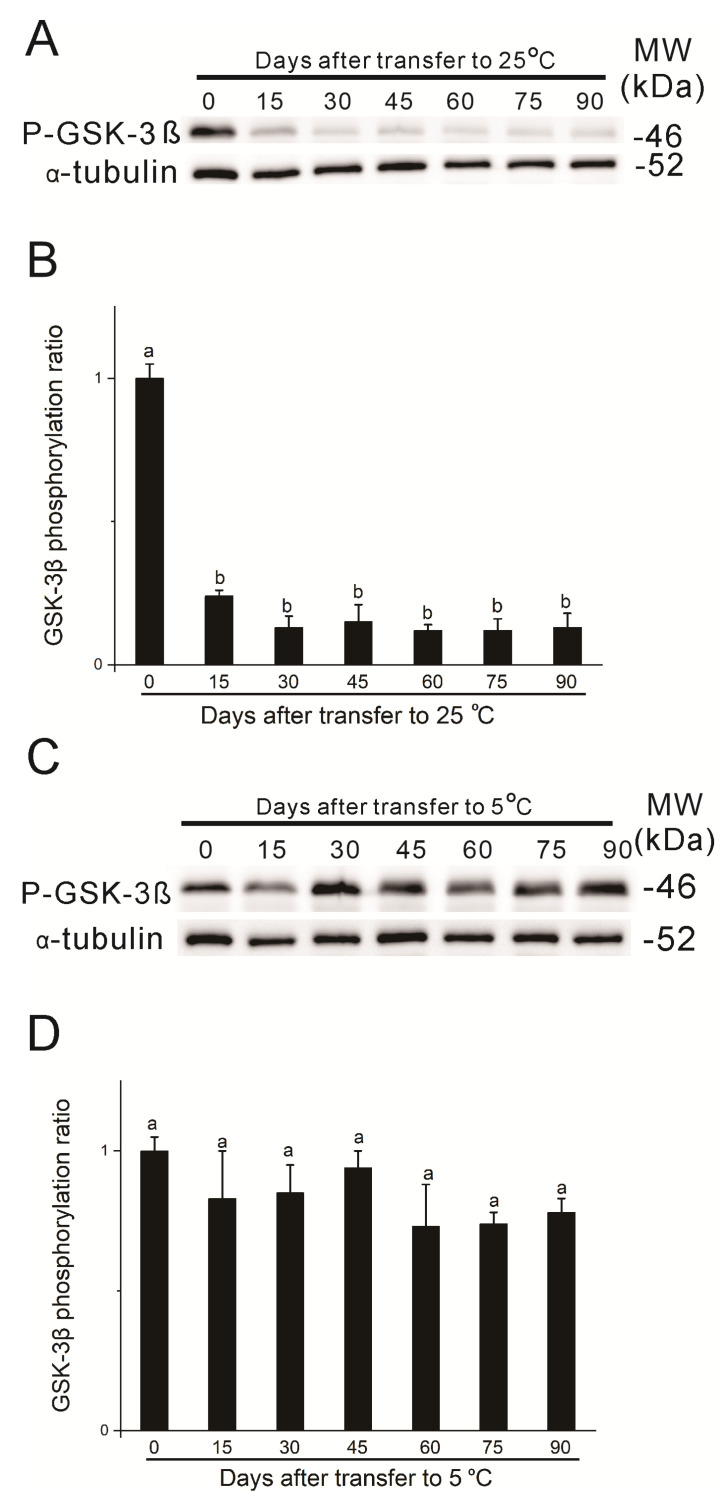
Temporal changes in phosphorylation levels of GSK-3β in diapause eggs incubated at 25 °C (**A**,**B**) and those exposed to 5 °C (**C**,**D**). Egg lysates from each treatment were prepared and subjected to immunoblot analysis with anti-phospho-GSK-3β (Ser9) (P-GSK-3β) and anti-α-tubulin (α-tubulin) antibodies. (**A**,**C**) Western blotting. Molecular weight (MW) markers are shown on the right side of the gel. Results shown in panels (**A**,**C**) are representative of four independent experiments. (**B**,**D**) Quantitative data from scans of Western blots. Data are expressed as fold changes over the first day before treatment after being normalized to the total amount of α-tubulin. Different letters above the bars indicate a significant difference (*n* = 4).

**Figure 6:**
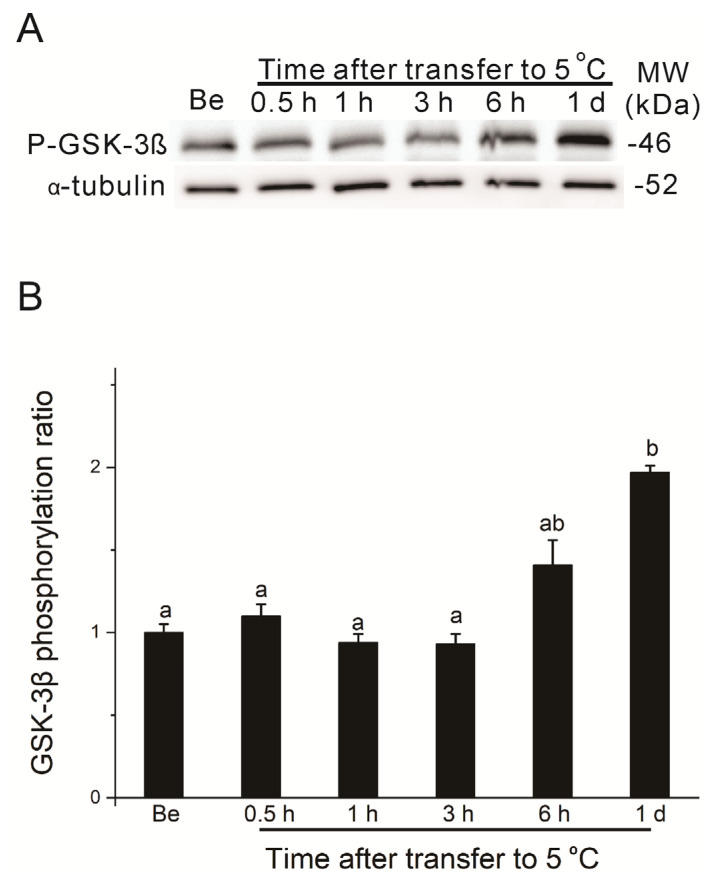
Time-dependent effects of chilling on phosphorylation levels of GSK-3β in silkworm eggs. Egg lysates from each treatment were prepared and subjected to immunoblot analysis with anti-phospho-GSK-3β (Ser9) (P-GSK-3β) and anti-α-tubulin (α-tubulin) antibodies. Diapause eggs were incubated at 25 °C for 15 days after oviposition and then were transferred to 5 °C for different time periods (from 0.5 h to 1 day). Be, before transfer to 5 °C. (**A**) Western blotting. Molecular weight (MW) markers are shown on the right side of the gel. Results shown in panel (**A**) are representative of four independent experiments. (**B**) Quantitative data from scans of Western blots. Data are expressed as fold changes over the controls (Be) after being normalized to the total amount of α-tubulin. Different letters above the bars indicate a significant difference (*n* = 4).

The above results clearly showed that *bombyxin-A6 and EPPase* gene expressions were consistently maintained at relatively higher levels during the long chilling period compared to those at 25 °C. To further confirm that chilling affects insulin signaling, we studied the phosphorylation of GSK-3β, a downstream component of insulin signaling [28,43], and compared the differential expressions between chilled eggs and eggs kept at 25 °C. As shown in Figure 5A,B, the phosphorylated GSK-3β level was high on the first day after oviposition, decreased sharply on day 15, and thereafter remained at very low levels for eggs kept at 25 °C. However, in chilled eggs, phosphorylated GSK-3β levels were maintained at relatively higher levels throughout the chilling period (Figure 5C,D). To examine how fast the phosphorylated GSK-3β level responded to chilling, we used eggs whose chilling began on day 15 after incubation at 25 °C after oviposition. As shown in Figure 6, upon chilling on day 15, the phosphorylated GSK-3β level increased as early as 6 h after eggs were exposed to 5 °C and had reached a high level by 1 day after chilling. This result demonstrated that chilling indeed enhanced insulin signaling, as reflected by activation of the GSK-3β phosphorylation, thus confirming that relatively higher insulin signaling exists in chilled eggs compared to eggs kept at 25 °C.

### 3.4. Effects of Chilling on Ecdysteroid Levels 

**Figure 7:**
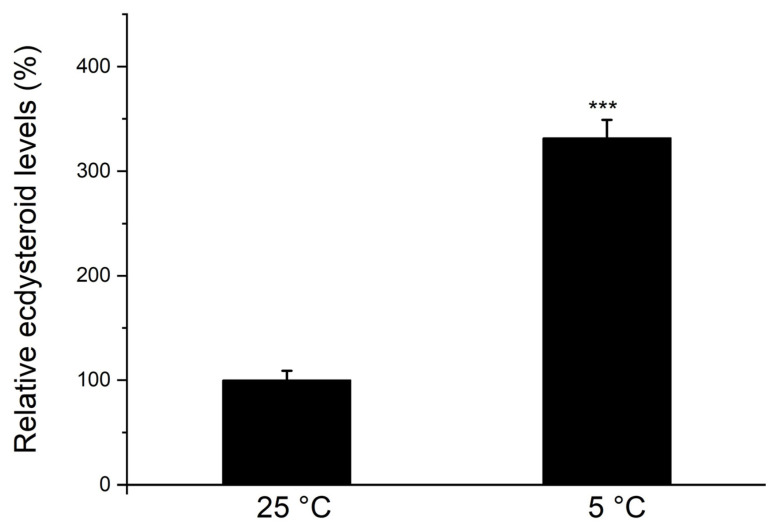
Comparison of ecdysteroid levels between eggs exposed to 5 °C and those incubated at 25 °C. After incubation at 25 °C for 2 days post-oviposition, diapause eggs were divided into two groups: one group was continually incubated to maintain diapause (25 °C), while the other group was exposed to 5 °C to break diapause (5 °C). Each group was maintained for 90 days, and ecdysteroid levels in egg extracts were determined with an EIA kit. Each data point is the mean ± SEM (*n* = 4). Asterisks indicate a significant difference (by Student’s *t*-test, *** *p* < 0.001).

We also studied the effects of chilling on ecdysteroid levels. Figure 7 shows that upon chilling on day 2 after oviposition, a higher ecdysteroid level was detected in chilled eggs on day 90 compared to eggs incubated at 25 °C. Because the antibody could not distinguish between 20-hydroxyecdysone and other ecdysteroids, this result likely represents the amounts of total ecdysteroids.

## 4. Discussion

**Figure 8:**
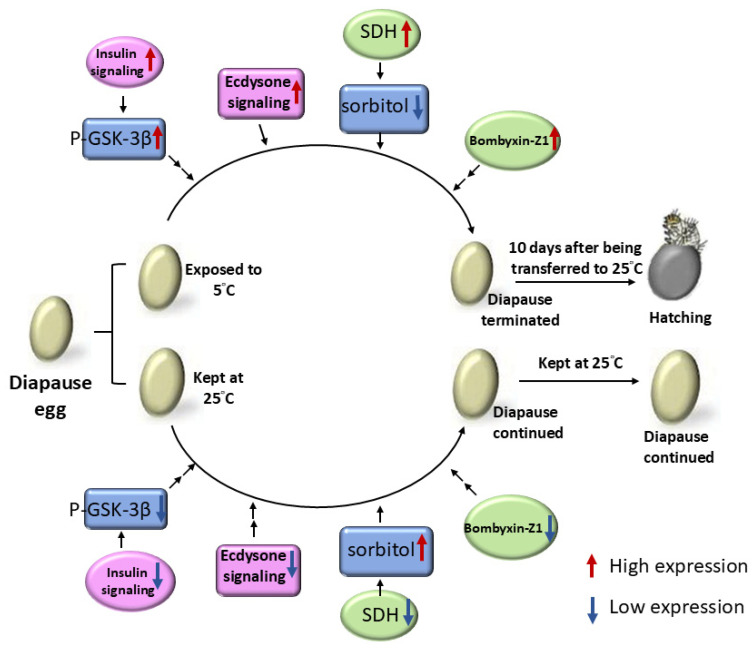
Diagrammatic representation of the proposed model showing the correlation between upregulation in insulin/ecdysone signaling and chilling-induced diapause termination in *B. mori* eggs. See text for details.

Results presented here clearly show that differential changing patterns exist in gene expression levels of *bombyxin-A6*, *-Z1*, *EPPase*, and *SDH2* between eggs exposed to 5 °C and those kept at 25 °C. In eggs kept at 25 °C, expression levels of all genes decreased to very low levels during the 3-month incubation period. However, in eggs exposed to 5 °C on day 2 after oviposition, changes in expression levels differed among the various genes. Expression levels of the *bombyxin-A6* and *EPPase* genes remained at constant, relatively high levels when eggs were exposed to 5 °C compared to those kept at 25 °C. Expression levels of *bombyxin-Z1* initially decreased upon exposure to 5 °C during the first 15 days after chilling and reached the lowest levels after 30 days. However, expression levels of *bombyxin-Z1* gradually increased between 45 and 90 days. For *SDH2* gene expression, the level was very low during the first stages of the chilling period; it then increased after 30 days of exposure to 5 °C and reached high levels between 45 and 90 days. In addition, our results showed that relatively higher GSK-3β phosphorylation levels were detected in chilled eggs compared to those kept at 25 °C, thus further confirming the stimulatory effect of chilling on insulin signaling. Although a previous study showed the changing pattern of *SDH2* gene expression upon chilling [36], which was confirmed in the present study, to our knowledge, this is the first study demonstrating differential temporal changes in *bombyxin-A6*, *-Z1*, and *EPPase* gene expressions between eggs exposed to 5 °C and those kept at 25 °C and clarifying the activating effects of chilling on insulin and ecdysone signaling, which are likely related to chilling-induced diapause termination in *B. mori* eggs (Figure 8).

As shown in Figure 2, expression levels of *bombyxin-A6* remained at constant, relatively high levels when eggs were exposed to 5 °C compared to those kept at 25 °C. This result indicated that relatively higher insulin signaling exists in chilled eggs compared to those kept at 25 °C. Higher expression levels of both *Tret1* and *Treh1* in chilled eggs compared to those kept at 25 °C also supported this assumption. Our present study showed that phosphorylation of GSK-3β, a key enzyme that regulates glycogen synthesis in response to insulin [43], remained at relatively higher levels in eggs exposed to 5 °C compared to those kept at 25 °C. Moreover, we further demonstrated that phosphorylation levels of GSK-3β increased as early as 6 h after eggs were exposed to 5 °C and remained at relatively higher levels the next day, clearly indicating that chilling enhanced insulin signaling. In *Drosophila*, evidence showed that stimulation of cold-sensing neurons activates insulin-producing cells, promotes synthesis and secretion of insulin-like peptides, and induces a larger body size, thus mimicking the effects of rearing the flies in cold temperatures [44]. Future research is needed to clarify the molecular link of chilling-induced insulin signaling with diapause termination in *Bombyx*.

Thus, it is suggested that compared to eggs kept at 25 °C, chilled eggs maintained relatively higher glucose metabolism, leading to sustained consumption of stored glycogen as fuel during the long chilling period. As the depletion of metabolic reserves was sustained and nutrient reserves reached a threshold, chilled eggs shifted toward mobilizing sorbitol to glucose to meet energetic demands. At this critical time, *SDH*2 gene expression began to increase after 30 days of exposure to 5 °C and reached high levels between 45 and 90 days. Previous studies showed parallel temporal changes between *SDH* gene expression and its enzymatic activity [16,17,36]. Thus, under the action of SDH, sorbitol is converted to glycogen. Subsequently, glycogen breaks down into glucose-1-phosphate and glucose, and glucose is ultimately utilized as an energy source for sustaining diapause and eventual termination of diapause in chilled eggs. From these results, it is reasonable to suppose that chilled eggs undergo slow development during the long chilling period, with sustained low metabolism. Similar slow development was reported in aphid embryonic diapause [45,46]. It was demonstrated that in *Acyrthosiphon pisum*, diapausing embryos showed evident cell division and leg growth during the diapause maintenance phase [47]. More recently, in *Ostrinia nubilalis*, evidence showed that diapausing larvae of the earlier emerging, bivoltine E-strain suppressed cell cycle progression less than in later emerging, univoltine Z-strain individuals, with a greater proportion of cells in the S phase during diapause [48].

It has been well demonstrated that mammalian cells do not generally take up nutrients in a cell-autonomous manner [49]. Nutrient acquisition is directed primarily by growth factor signaling [50]. Our recent studies demonstrated that nutrient uptake, as revealed by increased expressions of *Sts* and *Treh1*, appears to play a role in bombyxin-II or prothoracicotropic hormone (PTTH)-stimulated ecdysteroidogenesis of prothoracic glands in *B. mori* [29,39]. Considering the critical role of growth factors in stimulating nutrient uptake, it is reasonable to assume that glucose released under the action of SDH in chilled eggs might act as a signal molecule to trigger increases in *bombyxin-Z1* gene expression levels. Thus, after the occurrence of an increase in *SDH* gene expression levels at 30 days, *bombyxin-Z1* expression levels began to increase after 45 days of exposure to 5 °C and reached high levels after 60 to 90 days of exposure to 5 °C. We speculated that dramatically increased expressions of *SDH2* and *bombyxin-Z1* genes in chilled eggs might contribute to replenishing energy and maintaining nutrient homeostasis to the end of diapause. In *Drosophila*, it was well demonstrated that fat body signals remotely control brain neurosecretory cells to release insulin, thus playing a critical role in integrating hormonal and nutritional signals [51,52]. A previous study of *Helicoverpa armigera* showed that intermediates of the tricarboxylic acid (TCA) cycle released from fat bodies act in the brain, stimulating ecdysone biosynthesis in prothoracic glands, thus terminating pupal diapause [53]. It was further reported that matrix metalloproteinase-induced fat body cell dissociation of *H. armigera* promotes pupal development and moderately averts pupal diapause by enhancing lipid metabolism [54]. The yolk is the major internal nutritional supply on which most embryos rely [55]. In *B. mori*, yolk cell granulation and dissociation begin 45 days after exposure to 5 °C, coinciding with increased SDH2 expression [21]. We suggest that an evolutionarily conserved regulatory mechanism exists underpinning diapause termination from nutrient demands to growth factor signaling. In addition, it was interesting to note that *bombyxin-Z1* expression was related to both diapause initiation and chilling-induced diapause termination. A previous study showed that abruptly decreased *bombyxin-Z1* gene expression levels were detected in the very early diapause initiation stage [32]. In chilled eggs, *bombyxin-Z1* expression levels increased 45 days after exposure to 5 °C, which coincided with the initiation of diapause termination in chilled eggs. Moreover, our results showed that upon chilling, *bombyxin-A6* expression always remained at relatively high levels. This result indicated that different *bombyxin* genes showed stage-specific expressions during the long chilling period, clearly indicating that different bombyxins may play stage-specific roles during the embryonic diapause termination process in *B. mori*.

In addition, in diapause eggs of *B. mori*, it was reported that sorbitol plays a critical role in ensuring diapause, as its concentration must decrease for embryonic development to resume [12]. Utilization of sorbitol is controlled by the activation of SDH enzymatic activity in chilled eggs, which decreases the sorbitol concentration and eventually results in the termination of diapause and the resumption of development. However, eggs kept at 25 °C maintained very low insulin signaling and minimized glucose metabolism; thus, diapause was sustained, and no developmental resumption occurred during the first 90 days of the incubation period. In the current study, all gene expressions remained at very low levels in eggs kept at 25 °C. This result is consistent with previous studies, clearly indicating that diapause silences gene expressions [56]. It was previously reported that when diapause eggs were incubated at 25 °C for 400 days, they did not resume embryonic development [57], presumably due to an inability to utilize sorbitol. 

Moreover, expression levels of *EPPase* consistently remained at relatively higher levels in chilled eggs compared to those kept at 25 °C. We also detected relatively higher ecdysteroid levels and ecdysone signaling gene (*E75A*) expression levels in chilled eggs. We hypothesized that in eggs exposed to 5 °C, due to high EPPase expression, higher ecdysteroid levels, and ecdysone signaling, together with relatively higher insulin signaling, may coordinately contribute to the slow development, eventually leading to diapause termination. Currently, it is unclear whether the chilled eggs synthesize new ecdysteroids. Our future study will investigate the temporal changes in ecdysteroidogenesis during the long chilling period.

In conclusion, the results presented herein, which are summarized in Figure 8, not only showed that chilled eggs maintained relatively high levels of insulin and ecdysone signaling but further demonstrated different temporal changes in *bombyxin-Z1* and *SDH2* gene expression levels between eggs exposed to 5 °C and those kept at 25 °C. Our results also showed that chilling enhanced insulin signaling as reflected by GSK-3β phosphorylation levels as early as 6 h after eggs were exposed to 5 °C. These results suggest that relatively higher levels of both insulin and ecdysone signaling during the first 30 days after exposure to 5 °C in chilled eggs are likely related to a later increase in *SDH2* and *bombyxin-Z1* gene expression levels, which ultimately lead to decreased sorbitol levels and diapause termination of *B. mori*. 

## Data Availability

Data will be made available at the request of the corresponding author.

## Supplementary Materials

The following supporting information can be downloaded at https://www.mdpi.com/article/10.3390/insects15120989/s1, Table S1. List for the primers of qRT-PCR.
