# Supplementary material for: Upregulation of Insulin and Ecdysone Signaling in Relation to Diapause Termination in Bombyx mori Eggs Exposed to 5 °C
Source: Insects. 2024 Dec 12;15(12):989. doi: 10.3390/insects15120989 (PMC11678774; doi:10.3390/insects15120989)
Supplement: Supplementary file 1 [file insects-15-00989-s001.zip › insects-3325701-supplementary.pdf]

Table S1. qRT-PCR primer sequences

| Gene name | Accession No. | Forward primer | Reverse primer | Note |
|---|---|---|---|---|
| Rp49 | NM_001098282.1 | CAGGCGGTTCAAGGGTCAATAC | TGCTGGGCTCTTTCCACGA | Housekeeping gene |
| Bombyxin-II | NM_001128315.1 | GATGCCGTACTCGGAAGGAC | TACGACAGAAGCACGTCCAC | Insulin signaling genes |
| Bombyxin-Z1 | NM_001246356.1 | CTGTATTTCCTGATCGTTGTG | GTGTCATAGCAGAGTACCAT | |
| Bombyxin-Y1 | NM_001145324.1 | CGTACCTTGGCCAATCTCTGCAGC | GGCGACGCCATTCTCAGTCACG | |
| Tret1 | NM_001114872.1 | AATACCGGTGCCATTCTTCC | TTCCTCTTAACCAGCGCAGA | Metabolism-related genes |
| Treh1 | S73271.1 | CCCTGTGGATGAACGCAGTC | GCCAAGCGTTAGGGAAGTCC | |
| SDH2 | NM_001044127.1 | TGAGATTCCGCGCGTCC | GCAGCAGGACCTCATCTTCA | |
| Spo | NM_001111363.1 | TGGATAGGAACACTATTATCTT | AGACACAGCATGACTAAG | Ecdysone-related genes |
| E75A | AF332550.1 | GCTCCTCTTAATAGTATCA | AAGTAGAATCAACGAGAA | |
| EPPase | XM_038020193.1 | TTCATGACGCCCATCGAACT | CGTTACCGCCGTCTTTTTCG | |
